# Prognostic Significance of Disseminated Tumor Cells in Bone Marrow for Endometrial Carcinoma Patients

**DOI:** 10.3390/jcm13154489

**Published:** 2024-07-31

**Authors:** Léa Louise Volmer, Marcel Grube, Annika Rohner, Jessica Nell McAlpine, Aline Talhouk, Amy Lum, Sabine Matovina, Stefan Kommoss, Annette Staebler, Sara Yvonne Brucker, Christina Barbara Walter

**Affiliations:** 1 Department of Women’s Health, Tübingen University, 72074 Tübingen, Germanysara.brucker@med.uni-tuebingen.de (S.Y.B.);; 2 Women’s Hospital, Diakonie-Klinikum, 74523 Schwäbisch Hall, Germany; 3 Department of Gynecology and Obstetrics, Division of Gynecologic Oncology, University of British Columbia, Vancouver, BC V6T 1Z4, Canada; 4 Department of Pathology and Laboratory Medicine, University of British Columbia, Vancouver, BC V6T 1Z4, Canadaamylum@bccrc.ca (A.L.); 5 Institute of Pathology, Tübingen University, 72074 Tübingen, Germany

**Keywords:** endometrial cancer, ProMisE classification, disseminated tumor cells, molecular classification, minimal residual disease

## Abstract

**Background:** Until now, limited clinical significance had been reported for disseminated tumor cells (DTCs) in gynecologic malignancies. DTCs were previously reported not to be associated with established risk factors, L1CAM immunoreactivity, and outcome in endometrial carcinoma (EC). This study’s primary objective was to investigate potential correlations of DTCs in the bone marrow (BM) of EC patients with disease-related survival, and a secondary objective was to evaluate associations between molecular classification of EC and DTCs. **Methods:** Patients treated for primary EC at Tuebingen University women’s hospital between 2003 and 2016 were identified. A total of 402 patients with a complete set of BM cytology, molecular, and clinical data were evaluable. **Results:** DTC occurrence was distributed equally among all four molecular groups (*p* = 0.651). DTC positivity was associated with a less favorable disease-free survival (HR: 1.86, 95% CI: 1.03–3.36, *p* = 0.036) and progression-free survival (HR: 1.86, 95% CI: 1.01–3.44, *p* = 0.045). Presence of DTCs was associated with a higher frequency of distant disease recurrence (*p* = 0.017). **Conclusions:** In line with our previous findings, tumor cell dissemination is not associated with molecular features in our large cohort of primary EC patients. Since DTCs seem to be associated with survival and location of disease recurrence, further studies are needed to decisively define their role in EC survival.

## 1. Introduction

Micrometastasis is known to play a role in the prognosis of cancer patients. More precisely, the presence of disseminated tumor cells (DTCs) in the bone marrow of breast patients is associated with a poorer outcome [1] as well as with earlier locoregional and distant relapse [2,3]. In breast cancer, bone marrow can act as a niche to DTCs, where tumor cells can remain dormant and lead to disease recurrence even years after breast-cancer treatment [4,5].

DTCs do not only play an important role in gynecological cancers. Although most studies on this topic are currently being conducted on breast cancer, studies have produced promising initial results on other solid tumors. For example, a meta-analysis showed that the presence of DTCs in prostate cancer was associated with a poorer prognosis [6]. In colorectal carcinoma, there are also data showing that the presence of DTCs correlates significantly with PFS [7]. Similar data are also available for non-small cell lung carcinoma [8], pancreatic carcinoma [9], and esophageal carcinoma [10].

In ovarian cancer, micrometastasis in the form of circulating tumor cells (CTCs) has been shown to be associated with a poorer prognosis [11]. Similarly, DTCs can be detected in patients with gynecological malignancies [12]. However, their prognostic significance in gynecological cancers, especially in endometrial cancer, remains uncertain [13,14]. With the appearance of modern prognostic markers in endometrial carcinoma, our group could previously show that the presence of DTCs was not associated with L1CAM or histopathological risk factors [15], an established prognostic marker in endometrial carcinoma [16,17].

In the past decade, Cancer Genome Atlas (TCGA)-derived molecular markers have been identified, shown to be highly relevant in determining the prognosis of endometrial carcinoma [18,19]. Based on these findings, molecular classifiers were developed [20], confirmed [21], and externally validated [22] and implemented in international trials [23,24]. Even more recently, therapeutic decisions were based on the molecular classification of endometrial cancer [25,26], and thus molecular classification was implemented in guidelines [27,28].

It is the aim of this study to investigate potential associations of TCGA-derived molecular features such as *POLE*-mutation status, p53 abnormalities, or MMR deficiency and the presence of DTCs in the bone marrow of endometrial carcinoma patients.

## 2. Materials and Methods

### 2.1. Study Population

Patients treated for primary endometrial carcinoma at the Tuebingen University Women’s Hospital between 2003 and 2016 were identified. Clinical data were collected from patient charts. Follow-up data received from the Tuebingen University Hospital Clinical Cancer Registry were updated, allowing for the evaluation of disease-specific survival (DSS) in all cases. ESMO 2020 risk classifications were performed following published guidelines [27,29]. For disease progression patterns, the first location of disease recurrence or of metastasis was considered. Patients with a follow-up <6 months were excluded from survival analysis. Patients with initial FIGO IVB or FIGO IVA without curative locoregional therapy were excluded from analysis of disease progression pattern. For patient selection; see Supplementary Figure S1. All patients provided written informed consent into bone marrow aspiration and data analysis. The study protocol was approved by the local ethics committee (299/2017BO2, 26 October 2022).

### 2.2. DTC Detection

Bone marrow sampling was performed during surgery for endometrial carcinoma. All bone marrow samples were processed within 24 h. Mononuclear cells from the bone marrow were isolated by density centrifugation (Ficoll, 1.077 g/mL, Biochrom, Berlin, Germany). These cells were then spun down onto a glass slide (cytocentrifuge, Hettich, Tuttlingen, Germany) and fixed in 4% formalin. The obtained cytospins were stained using the DAKO Autostainer (DAKO, Glostrup, Denmark). Mouse monoclonal antibodies A45-B/B3 directed against pancytokeratin (Micromet, Munich, Germany) were used. For cytokeratin staining, two slides with each 1.5 × 10^6^ cells per patient were evaluated, according to the consensus recommendations for standardized tumor cell detection [30]. Each batch of samples was analyzed together with leukocytes from healthy volunteers as negative controls and the human breast cancer cell lines MCF7 and SKBR3 as positive controls. DTC positivity was defined as at least one pancytokeratin-positive cell with typical cell morphology per 3.0 × 10^6^ cells. In breast cancer, higher percentages of patients with at least ≥2 DTCs/1.5 × 10^6^ mononuclear cells were seen in the more aggressive triple-negative subtype [31]; therefore, a second cut-off of ≥2 DTCs/1.5 × 10^6^ mononuclear cells was defined to evaluate similar differences between EC molecular subtypes.

### 2.3. Molecular Classification

Only patients with available information on endometrial carcinoma molecular classifiers were included in this study. For these patients, the prognostic relevance of molecular classification was validated in a prior study [22]. Molecular subgroups were assigned according to the Proactive Molecular Risk Classifier for Endometrial Cancer (ProMisE) [20]: patients were classified as *POLE* mutated (*POLE*mut), mismatch-repair deficient (MMRd), p53-abnormal (p53abn), or no specific molecular profile (NSMP).

### 2.4. Statistical Analysis

Correlations between DTC status and patient’s characteristics, as well as molecular classification, were evaluated using the chi square test. For survival analysis, duration from diagnosis to disease progression (PFS) and to death of endometrial carcinoma (disease-specific survival, DSS) or death of any cause (overall survival, OS) were calculated separately. If no event occurred, data were censored at timing of last follow up. Patients with a follow-up duration <6 months were excluded from survival analysis. Kaplan–Meier curves were plotted and compared using the log rank test. Median follow up was calculated with the reverse Kaplan–Meier method. Cases with primary-stage FIGO IV or without complete radical resection at time of first diagnosis were excluded from analysis of location of disease progression. All statistical analyses were performed using JMP16 (SAS^®^). The significance level was set at *p* < 0.05.

## 3. Results

### 3.1. Patient Characteristics

A total of 402 patients treated for endometrial cancer at Tuebingen Women’s University Hospital between 2003 and 2016 were included in this study. Patient age at diagnosis ranged from 30 to 87 years (median 64.8). Patient body-mass-index (BMI) ranged from 15.2 to 61.7 (median 27.7). The majority of cases (339/402, 84.3%) were type I endometrial carcinomas (endometrioid histology), and the remaining type II cases (63/402, 15.7%) were diagnosed with serous (32/63), clear-cell (6/63), or mixed histology (25/63). Additionally, 243 (60.4%) tumors were G1, 68 (16.9%) were G2, and 91 (22.6%) were G3. Further, 251 (62.4%) patients were diagnosed with FIGO stage IA disease, and the remaining cases were FIGO stage IB (76, 18.9%), FIGO stage II (21, 5.2%), FIGO stage III (48, 11.9%), and FIGO stage IV (6, 1.4%). Applying the 2020 ESMO risk stratification criteria as mentioned above, 211 (57.0%) carcinomas were low-risk, 64 (17.3%) were intermediate-risk, 30 (8.1%) were high-intermediate-risk, and 61 (17.0%) were high-risk, according to the 2020 ESMO guidelines. Myoinvasion was evaluated in 386 cases, and invasion >50% of myometrium was detected in 125 cases (32.4%). Lymph nodes were positive in 41/367 cases (11.1%).

Overall, DTCs were detected in 71 (17.7%) patients. Regarding patient’s characteristics, no significant difference was found between DTC-positive and -negative patients (see Table 1). Higher numbers of DTCs (≥2 DTCs/1.5 × 10^6^ mononuclear cells) were detected in 9 (2.3%) of patients.

### 3.2. Molecular Classification

ProMisE molecular classification revealed 40 *POLE*mut (10.0%), 103 MMRd (25,6%), 52 p53-abnormal (12.9%), and 207 (51.5%) tumors with no specific molecular profile (NSMP).

DTC occurrence was distributed equally among molecular groups (see Table 2). In patients with a p53abn subtype, a higher percentage of cases showed higher numbers of DTCs (≥2 DTCs/1.5 × 10^6^ mononuclear cells; 5.8% vs. overall 2.2% in all patients, *p* = 0.423).

### 3.3. Survival Analysis

The median follow-up time was 120 months (6–230 months). Follow-up information and DTC status were available for 394 cases.

The predictive value of molecular groups was previously validated in this cohort [22] and confirmed with updated follow-up: patients showing a p53 mutation showed the most impaired prognosis, with 17/46 disease-related deaths (HR: 3.92, 95% CI: 2.42–6.33, *p* < 0.001), whereas patients with a POLE mutation showed no disease-related fatal outcomes (see Supplementary Figure S2).

**Figure 1:**
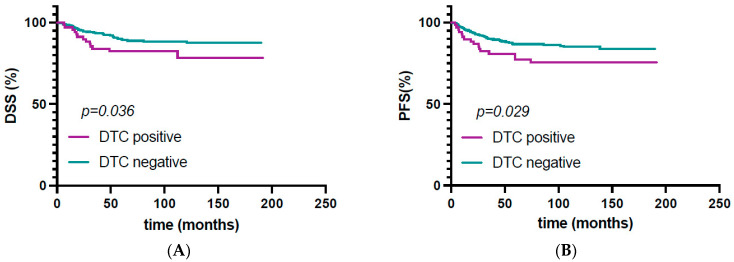
Univariate survival analysis by disseminated tumor cell (DTC) status. Kaplan–Maier plots of (**A**) disease-specific survival (DSS) and (**B**) progression-free survival for DTC detection in bone marrow samples of patients with endometrial carcinoma.

Presence of DTCs was not significantly associated with worse overall survival (*p* = 0.069). DTC-positive patients showed a poorer progression-free survival (HR: 1.91, 95% CI: 1.06–3.45, *p* = 0.029); see Figure 1. Furthermore, DTC positivity was associated with an impaired disease-specific survival (HR: 1.86, 95% CI: 1.03–3.36, *p* = 0.036); see Figure 1. Disease-specific fatal outcome was observed in 14/70 (20.0%) DTC-positive patients, whereas 36/324 (9.1%) of DTC-negative patients had lethal outcomes due to endometrial carcinoma.

**Figure 2:**
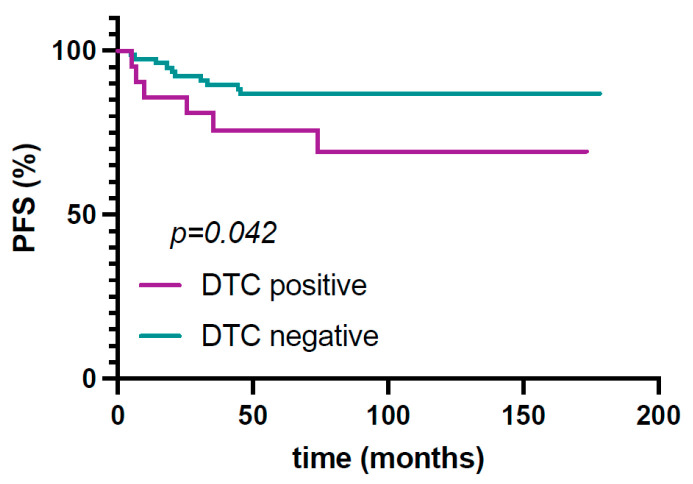
Univariate survival analysis by disseminated tumor cell (DTC)-status. Kaplan–Maier plot of progression-free survival for DTC detection in bone marrow samples of endometrial carcinoma patients classified as MMRd.

For overall and disease-specific survival, no significant association between DTCs and survival was found in a specific molecular group. In patients with a *POLE*mut, p53abm, and NSMP molecular subtype, DTC detection was not significantly associated with progression-free survival. However, in patients with an MMRd subtype, the detection of DTC was associated with a poorer progression-free survival (HR: 2.92, 95% CI: 1.04–8.20, *p* = 0.042); see Figure 2.

### 3.4. Disease Progression Pattern

Disease progression occurred in 56 cases (13.9%). Full staging information for the location of disease progression was available for 43 patients with initial complete resection and/or initial stage < FIGO IV, revealing 16 (37.2%) cases of locoregional progression and 27 (62.8%) cases of distant progression.

Molecular classification was not significantly associated with distant or locoregional disease progression; see Table 3.

Disease progression was also not significantly associated with further histopathological parameters; see Supplementary Table S1.

The pattern of disease progression was associated with the presence of DTCs: disease progression occurred more frequently in the form of distant metastasis in DTC-positive patients (84.6%), while more locoregional relapses were seen in DTC-negative patients (53.3%, *p* = 0.042; see Table 4).

## 4. Discussion

TCGA-derived molecular classification of endometrial carcinoma has fundamentally changed our understanding of the disease and led to a new risk stratification [32]. With differences in prognosis between molecular groups, the call for individualized therapeutic decisions is becoming louder. In response to this, international trials on individualization of adjuvant radiation and systemic therapy based on histopathological and molecular features have been designed [23,24]. Further stratification of molecular groups could already be achieved, for example, via assessment of grading and estrogen receptor status in NSMP endometrial carcinomas [33]. Also, recent studies have confirmed the prognostic significance of MRD in the form of DTCs in breast cancer [34]. In search for an even more refined risk stratification for endometrial carcinoma patients and taking a longer follow-up duration into account, a possible impact of minimal residual disease (MRD) on disease-related prognosis was investigated in this cohort. With full information on molecular classification available in this cohort, a possible association between molecular groups and the presence of DTCs was evaluated.

In this cohort, the detection of DTCs was homogenous throughout all molecular groups, with 17.5% DTC-positive patients even in the prognostically highly favorable *POLE*mut group. Similarly to these results, our group could previously show that the presence of DTCs was not associated with L1CAM [15], another established prognostic marker in endometrial carcinoma [16,17].

High amounts of DTCs (≥2 DTCs/1.5 × 10^6^ mononuclear cells) were found at a higher percentage in high-risk p53abn patients (5.5%, *p* = 0.423). However, with very few such cases (overall 9 cases, 2.2% of patients), no sufficient conclusion about survival or association with other pathological or clinical parameters can be drawn in patients with high amounts of DTCs. Comparison of DTC detection rates with those from other cohorts is not feasible, since no data are available to this topic. Liquid biopsy in general is rarely performed on patients with gynecological cancer, and published data range from 7% to 75% detection of circulating tumor cells in the peripheral blood of endometrial cancer patients [35,36,37].

DTC-positive and -negative patients did not differ by histopathological or biometrical characteristics. These results are opposed to those in breast cancer patients, where DTCs are more frequently detected in patients with a higher risk profile [31,34]. The differences between DTC prevalence and its impact on survival in molecular groups may be reflected by the biological heterogeneity of endometrial carcinoma molecular subtypes themselves. For example, *POLE*mut tumors are stipulated to be immunogenic due to their high mutational load [38]. DTCs derived from this hypermutated subtype may develop the same way as cells in the primary tumor and very seldom lead to relapse or metastases.

In this cohort from a large oncological center, disease progression occurred in around 14% of cases overall. The frequency and location of disease progression for this cohort concur with prior reports [39,40,41]. DTC detection was found to be associated with an impaired DSS and PFS. These results are in contrast with previously reported survival data from our group [13,15]; however, this cohort differs from the previous study and in the latest study showed a tendency towards an impact of DTC detection on survival (DSS (*p* = 0.14)) [15]. Another study previously showed an association between CTCs and a worse overall survival in gynecological malignancies [42]. Furthermore, similar results have been reported in colorectal cancer, where the presence of DTCs in the BM was associated with an adverse PFS [43].

Furthermore, the presence of DTCs in the BM of endometrial cancer patients was found to be associated with more frequent distant disease progression. Therefore, DTCs in the BM may act as a surrogate parameter for tumor cell propagation and as a predictor for disease recurrence, similarly to breast cancer [34]. Correlation of these results with other studies is not adequately feasible, since there are very few prior studies on micrometastasis and disease progression in endometrial cancer. One study found no association between CTCs and distant metastases; however, the cohort comprised only fifteen CTC-positive patients [44].

Functional assays investigating the biological behavior of DTCs isolated from EC patients while correlating results with the molecular subtype may help understand the impact of DTCs on EC survival. A method of DTC isolation offering more possibilities for molecular analyses of DTCs may be used in future studies [45].

In conclusion, DTC detection is independent from classical risk parameters, but also from molecular classification, while being relevant for disease-related survival and location of disease progression. This raises the question of whether tumor cell dissemination may define another independent risk parameter for endometrial carcinoma patients. Further studies with larger cohorts and molecular characterization of DTCs are needed to validate these findings and to identify DTCs with metastatic potential before implications for therapeutic decisions may be issued.

## Figures and Tables

**Table 1:** Patient characteristics by disseminated tumor cell (DTC) status.

	Total	DTC-Positive	DTC-Negative	*p*-Value *
	n	n (%)	n (%)	
Total	402	71 (17.7)	331 (82.3)	
FIGO stage (2018)				
IA	251	44 (17.5)	207 (82.5)	0.501
IB	76	12 (15.8)	64 (84.2)	
II	21	3 (14.3)	18 (85.7)	
III	47	10 (21.3)	37 (78.7)	
IV	7	2 (28.6)	5 (71.4)	
Type				
I	339	59 (17.4)	280 (82.6)	0.516
II	63	12 (19.0)	51 (81.0)	
Grade				
G1	243	43 (17.7)	200 (82.3)	0.933
G2	68	11 (16.2)	57 (13.8)	
G3	91	17 (18.7)	74 (81.3)	
Myoinvasion				
no	100	22 (22.0)	78 (78.0)	0.557
<50%	161	27 (16.8)	134 (83.2)	
>50%	125	22 (17.6)	103 (82.4)	
Lymph nodes				
Positive	41	7 (17.0)	34 (82.9)	0.797
Negative	326	61 (18.7)	265 (81.3)	
BMI				
<25	133	22 (16.5)	111 (83.5)	0.667
≥25	257	47 (18.3)	210 (81.7)	
Median age	-	67.3	64.2	0.115
ESMO 2020 risk group				
Low	211	44 (20.9)	167 (79.1)	0.349
Intermediate	64	7 (10.9)	57 (89.1)	
High-intermediate	30	4 (13.3)	26 (86.7)	
High	59	11 (18.6)	48 (81.4)	
Advanced metastatic	6	2 (33.3)	4 (66.7)	

* chi-square test.

**Table 2:** Molecular classification by DTC status.

	Total	DTC-Positive n (%)	*p*-Value *	≥2 DTCs/1.5 × 10^6^ Cells n (%)	*p*-Value *
All patients	402	71 (17.7)		9 (2.2)	
Molecular subtype			0.651		0.423
*POLE*mut	40	7 (17.5)		1 (2.5)	
p53abn	52	11 (21.2)		3 (5.8)	
MMRd	103	21 (20.4)		2 (1.9)	
NSMP	207	32 (15.5)		3 (1.5)	

* chi square test.

**Table 3:** Disease progression pattern by molecular subtype.

	Total	Distant Recurrence n (%)	Locoregional Recurrence n (%)	*p*-Value *
All patients	43	27 (62.8)	16 (37.2)	
Molecular subtype				0.123
*POLE*mut	1	1 (100.0)	0 (0.0)	
p53abn	12	10 (83.3)	2 (16.7)	
MMRd	14	9 (64.3)	5 (35.7)	
NSMP	16	7 (43.8)	9 (56.2)	

* chi-square test.

**Table 4:** Disease progression pattern by DTC status.

	Total	DTC-Positive n (%)	DTC-Negative n (%)	*p*-Value *
All patients	43	13 (30.2)	30 (69.7)	
Disease progression				0.042
Locoregional	16	2 (12.5)	14 (87.5)	
Distant	27	11 (40.7)	16 (59.3)	

* chi square test.

## Data Availability

The data presented in this study are available upon request from the corresponding authors.

## Supplementary Materials

The following supporting information can be downloaded at: https://www.mdpi.com/article/10.3390/jcm13154489/s1, Figure S1: flowchart summarizing the patient selection process; Figure S2: Univariate survival analysis by molecular classification. Kaplan–Maier plot of (A) overall survival, (B) disease-specific survival (DSS) and (C) progression-free survival (PFS) for molecular groups in endometrial carcinoma patients; Figure S3: ProMisE algorithm; Table S1: Patient characteristics by pattern of disease progression

## References

[1] Wiedswang G., Borgen E., Karesen R., Kvalheim G., Nesland J.M., Qvist H., Schlichting E., Sauer T., Janbu J., Harbitz T. (2003). Detection of isolated tumor cells in bone marrow is an independent prognostic factor in breast cancer. J. Clin. Oncol..

[2] Hartkopf A.D., Brucker S.Y., Taran F.A., Harbeck N., von Au A., Naume B., Pierga J.Y., Hoffmann O., Beckmann M.W., Ryden L. (2019). International pooled analysis of the prognostic impact of disseminated tumor cells from the bone marrow in early breast cancer: Results from the PADDY study. Cancer Res..

[3] Braun S., Vogl F.D., Naume B., Janni W., Osborne M.P., Coombes R.C., Schlimok G., Diel I.J., Gerber B., Gebauer G. (2005). A pooled analysis of bone marrow micrometastasis in breast cancer. N. Engl. J. Med..

[4] Chen F., Han Y., Kang Y. (2021). Bone marrow niches in the regulation of bone metastasis. Br. J. Cancer.

[5] Pantel K., Alix-Panabieres C. (2014). Bone marrow as a reservoir for disseminated tumor cells: A special source for liquid biopsy in cancer patients. Bonekey Rep..

[6] Ma X., Xiao Z., Li X., Wang F., Zhang J., Zhou R., Wang J., Liu L. (2014). Prognostic role of circulating tumor cells and disseminated tumor cells in patients with prostate cancer: A systematic review and meta-analysis. Tumour Biol..

[7] Lindemann F., Schlimok G., Dirschedl P., Witte J., Riethmuller G. (1992). Prognostic significance of micrometastatic tumour cells in bone marrow of colorectal cancer patients. Lancet.

[8] Pantel K., Izbicki J., Passlick B., Angstwurm M., Haussinger K., Thetter O., Riethmuller G. (1996). Frequency and prognostic significance of isolated tumour cells in bone marrow of patients with non-small-cell lung cancer without overt metastases. Lancet.

[9] Effenberger K.E., Schroeder C., Eulenburg C., Reeh M., Tachezy M., Riethdorf S., Vashist Y.K., Izbicki J.R., Pantel K., Bockhorn M. (2012). Disseminated tumor cells in pancreatic cancer-an independent prognosticator of disease progression and survival. Int. J. Cancer.

[10] Vashist Y.K., Effenberger K.E., Vettorazzi E., Riethdorf S., Yekebas E.F., Izbicki J.R., Pantel K. (2012). Disseminated tumor cells in bone marrow and the natural course of resected esophageal cancer. Ann. Surg..

[11] Giannopoulou L., Kasimir-Bauer S., Lianidou E.S. (2018). Liquid biopsy in ovarian cancer: Recent advances on circulating tumor cells and circulating tumor DNA. Clin. Chem. Lab. Med..

[12] Banys M., Solomayer E.F., Becker S., Krawczyk N., Gardanis K., Staebler A., Neubauer H., Wallwiener D., Fehm T. (2009). Disseminated tumor cells in bone marrow may affect prognosis of patients with gynecologic malignancies. Int. J. Gynecol. Cancer.

[13] Walter C.B., Taran F.A., Wallwiener M., Rothmund R., Kraemer B., Krawczyk N., Blassl C., Melcher C., Wallwiener D., Fehm T. (2014). Prevalence and prognostic value of disseminated tumor cells in primary endometrial, cervical and vulvar cancer patients. Future Oncol..

[14] Fehm T., Becker S., Bachmann C., Beck V., Gebauer G., Banys M., Wallwiener D., Solomayer E.F. (2006). Detection of disseminated tumor cells in patients with gynecological cancers. Gynecol. Oncol..

[15] Kommoss S., Hartkopf A.D., Kramer B., Bunz A.K., Grevenkamp F., Kommoss F., Pasternak J., Arbabi S.M., Wallwiener M., Staebler A. (2017). Disseminated tumor cells are not associated with established risk factors, L1CAM immunoreactivity and outcome in endometrial carcinoma. J. Cancer Res. Clin. Oncol..

[16] Coll-de la Rubia E., Martinez-Garcia E., Dittmar G., Gil-Moreno A., Cabrera S., Colas E. (2020). Prognostic Biomarkers in Endometrial Cancer: A Systematic Review and Meta-Analysis. J. Clin. Med..

[17] Corrado G., Laquintana V., Loria R., Carosi M., de Salvo L., Sperduti I., Zampa A., Cicchillitti L., Piaggio G., Cutillo G. (2018). Endometrial cancer prognosis correlates with the expression of L1CAM and miR34a biomarkers. J. Exp. Clin. Cancer Res..

[18] Cancer Genome Atlas Research N., Kandoth C., Schultz N., Cherniack A.D., Akbani R., Liu Y., Shen H., Robertson A.G., Pashtan I., Shen R. (2013). Integrated genomic characterization of endometrial carcinoma. Nature.

[19] Piulats J.M., Guerra E., Gil-Martin M., Roman-Canal B., Gatius S., Sanz-Pamplona R., Velasco A., Vidal A., Matias-Guiu X. (2017). Molecular approaches for classifying endometrial carcinoma. Gynecol. Oncol..

[20] Talhouk A., McConechy M.K., Leung S., Li-Chang H.H., Kwon J.S., Melnyk N., Yang W., Senz J., Boyd N., Karnezis A.N. (2015). A clinically applicable molecular-based classification for endometrial cancers. Br. J. Cancer.

[21] Talhouk A., McConechy M.K., Leung S., Yang W., Lum A., Senz J., Boyd N., Pike J., Anglesio M., Kwon J.S. (2017). Confirmation of ProMisE: A simple, genomics-based clinical classifier for endometrial cancer. Cancer.

[22] Kommoss S., McConechy M.K., Kommoss F., Leung S., Bunz A., Magrill J., Britton H., Kommoss F., Grevenkamp F., Karnezis A. (2018). Final validation of the ProMisE molecular classifier for endometrial carcinoma in a large population-based case series. Ann. Oncol..

[23] van den Heerik A., Horeweg N., Nout R.A., Lutgens L., van der Steen-Banasik E.M., Westerveld G.H., van den Berg H.A., Slot A., Koppe F.L.A., Kommoss S. (2020). PORTEC-4a: International randomized trial of molecular profile-based adjuvant treatment for women with high-intermediate risk endometrial cancer. Int. J. Gynecol. Cancer.

[24] Consortium R.R. (2022). Refining adjuvant treatment in endometrial cancer based on molecular features: The RAINBO clinical trial program. Int. J. Gynecol. Cancer.

[25] O’Malley D.M., Bariani G.M., Cassier P.A., Marabelle A., Hansen A.R., De Jesus Acosta A., Miller W.H., Safra T., Italiano A., Mileshkin L. (2022). Pembrolizumab in Patients With Microsatellite Instability-High Advanced Endometrial Cancer: Results From the KEYNOTE-158 Study. J. Clin. Oncol..

[26] Mirza M.R., Chase D.M., Slomovitz B.M., dePont Christensen R., Novak Z., Black D., Gilbert L., Sharma S., Valabrega G., Landrum L.M. (2023). Dostarlimab for Primary Advanced or Recurrent Endometrial Cancer. N. Engl. J. Med..

[27] Concin N., Matias-Guiu X., Vergote I., Cibula D., Mirza M.R., Marnitz S., Ledermann J., Bosse T., Chargari C., Fagotti A. (2021). ESGO/ESTRO/ESP guidelines for the management of patients with endometrial carcinoma. Radiother. Oncol..

[28] Emons G., Erdogan S., Die L. (2022). Neuerungen in der aktualisierten S3-Leitlinie Endometriumkarzinom. Forum.

[29] Colombo N., Creutzberg C., Amant F., Bosse T., Gonzalez-Martin A., Ledermann J., Marth C., Nout R., Querleu D., Mirza M.R. (2016). ESMO-ESGO-ESTRO Consensus Conference on Endometrial Cancer: Diagnosis, Treatment and Follow-up. Int. J. Gynecol. Cancer.

[30] Fehm T., Braun S., Muller V., Janni W., Gebauer G., Marth C., Schindlbeck C., Wallwiener D., Borgen E., Naume B. (2006). A concept for the standardized detection of disseminated tumor cells in bone marrow from patients with primary breast cancer and its clinical implementation. Cancer.

[31] Volmer L., Koch A., Matovina S., Dannehl D., Weiss M., Welker G., Hahn M., Engler T., Wallwiener M., Walter C.B. (2022). Neoadjuvant Chemotherapy of Patients with Early Breast Cancer Is Associated with Increased Detection of Disseminated Tumor Cells in the Bone Marrow. Cancers.

[32] McCluggage W.G., Singh N., Gilks C.B. (2022). Key changes to the World Health Organization (WHO) classification of female genital tumours introduced in the 5th edition (2020). Histopathology.

[33] Jamieson A., Huvila J., Chiu D., Thompson E.F., Scott S., Salvador S., Vicus D., Helpman L., Gotlieb W., Kean S. (2023). Grade and Estrogen Receptor Expression Identify a Subset of No Specific Molecular Profile Endometrial Carcinomas at a Very Low Risk of Disease-Specific Death. Mod. Pathol..

[34] Hartkopf A.D., Brucker S.Y., Taran F.A., Harbeck N., von Au A., Naume B., Pierga J.Y., Hoffmann O., Beckmann M.W., Ryden L. (2021). Disseminated tumour cells from the bone marrow of early breast cancer patients: Results from an international pooled analysis. Eur. J. Cancer.

[35] Kiss I., Kolostova K., Matkowski R., Jedryka M., Czekanski A., Pavlasek J., Bobek V. (2018). Correlation Between Disease Stage and the Presence of Viable Circulating Tumor Cells in Endometrial Cancer. Anticancer. Res..

[36] Bogani G., Liu M.C., Dowdy S.C., Cliby W.A., Kerr S.E., Kalli K.R., Kipp B.R., Halling K.C., Campion M.B., Mariani A. (2015). Detection of circulating tumor cells in high-risk endometrial cancer. Anticancer. Res..

[37] Klein A., Fishman A., Zemer R., Zimlichman S., Altaras M.M. (2000). Detection of tumor circulating cells by cytokeratin 20 in the blood of patients with endometrial carcinoma. Gynecol. Oncol..

[38] van Gool I.C., Eggink F.A., Freeman-Mills L., Stelloo E., Marchi E., de Bruyn M., Palles C., Nout R.A., de Kroon C.D., Osse E.M. (2015). POLE Proofreading Mutations Elicit an Antitumor Immune Response in Endometrial Cancer. Clin. Cancer Res..

[39] Fujimoto T., Nanjyo H., Fukuda J., Nakamura A., Mizunuma H., Yaegashi N., Sugiyama T., Kurachi H., Sato A., Tanaka T. (2009). Endometrioid uterine cancer: Histopathological risk factors of local and distant recurrence. Gynecol. Oncol..

[40] Creutzberg C.L., van Putten W.L., Koper P.C., Lybeert M.L., Jobsen J.J., Warlam-Rodenhuis C.C., De Winter K.A., Lutgens L.C., van den Bergh A.C., van der Steen-Banasik E. (2003). Survival after relapse in patients with endometrial cancer: Results from a randomized trial. Gynecol. Oncol..

[41] Kurra V., Krajewski K.M., Jagannathan J., Giardino A., Berlin S., Ramaiya N. (2013). Typical and atypical metastatic sites of recurrent endometrial carcinoma. Cancer Imaging.

[42] Kiss I., Kolostova K., Pawlak I., Bobek V. (2020). Circulating tumor cells in gynaecological malignancies. J. BUON.

[43] Wu P., Tang R.N., Zou J.H., Wang F.C. (2012). The prognostic role of disseminated tumor cells detected in peripheral blood and bone marrow of colorectal cancer. Hepatogastroenterology.

[44] Ni T., Sun X., Shan B., Wang J., Liu Y., Gu S.L., Wang Y.D. (2016). Detection of circulating tumour cells may add value in endometrial cancer management. Eur. J. Obstet. Gynecol. Reprod. Biol..

[45] Pillai S.G., Siddappa C.M., Ma C., Snider J., Kaushal M., Watson M.A., Aft R. (2021). A microfluidic-based filtration system to enrich for bone marrow disseminated tumor cells from breast cancer patients. PLoS ONE.

