# Supplementary material for: Prognostic Significance of Disseminated Tumor Cells in Bone Marrow for Endometrial Carcinoma Patients
Source: J Clin Med. 2024 Jul 31;13(15):4489. doi: 10.3390/jcm13154489 (PMC11313439; doi:10.3390/jcm13154489)
Supplement: Supplementary file 1 [file jcm-13-04489-s001.zip › Supplementary figure 3.pdf]

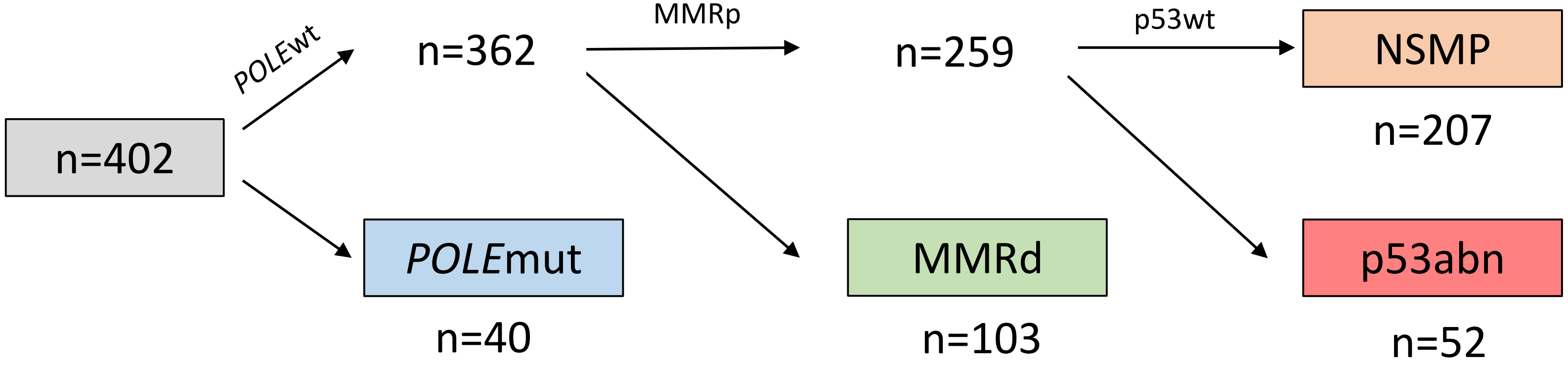

Supplementary figure 2:
ProMisE algorithm: samples first were assessed via immunohistochemistry (IHC) for the presence of mismatch repair proteins PMS2 and MSH6 with loss/deficient tumors categorized as MMRd (n= 103). Next, tumors were assessed for mutations in polymerase-E exonuclease domain, exons 9–14 (*POLE*mut, n = 40), and finally for aberrant expression of p53 by IHC yielding p53abn (n = 52) and p53wt (NSMP, n=207).
