# Supplementary material for: Prognostic Significance of Disseminated Tumor Cells in Bone Marrow for Endometrial Carcinoma Patients
Source: J Clin Med. 2024 Jul 31;13(15):4489. doi: 10.3390/jcm13154489 (PMC11313439; doi:10.3390/jcm13154489)
Supplement: Supplementary file 1 [file jcm-13-04489-s001.zip › Supplementary table 1.pdf]

Supplementary table 1: Patient characteristics by pattern of disease progression

	Total	distant progression	locoregional progression	p-value*
	n	n (%)	n (%)	
Total	45	28 (62.2)	17 (37.8)	
FIGO stage (2018)				
I	20	9 (18.0)	11 (92.0)	0.267
II	4	3 (75.0)	1 (15.0)	
III	19	15 (79.0)	4 (21.0)	
Type				
I	30	17 (56.7)	13 (43.3)	0.240
II	9	7 (77.8)	2 (22.2)	
Grade				
G1	13	5 (38.5)	8 (61.5)	0.060
G2	7	4 (57.1)	3 (72.9)	
G3	25	19 (76.0)	6 (24.0)	
Myoinvasion				
no	7	5 (71.4)	2 (28.6)	0.099
<50%	9	6 (66.7)	3 (33.3)	
>50%	26	19 (73.1)	7 (26.9)	
Lymph nodes				
positive	14	11 (78.6)	3 (21.4)	0.263
negative	26	16 (61.5)	10 (38.5)	
ESMO 2020 risk group				
low	8	4 (50.0)	4 (50.0)	0.561
intermediate	4	2 (50.0)	2 (50.0)	
high-intermediate	6	4 (66.7)	2 (33.3)	
high	20	15 (75.0)	5 (25.0)	

*chi-square test
