# Supplementary figures and images for: Prognostic Significance of Disseminated Tumor Cells in Bone Marrow for Endometrial Carcinoma Patients
Source: J Clin Med. 2024 Jul 31;13(15):4489. doi: 10.3390/jcm13154489 (PMC11313439; doi:10.3390/jcm13154489)

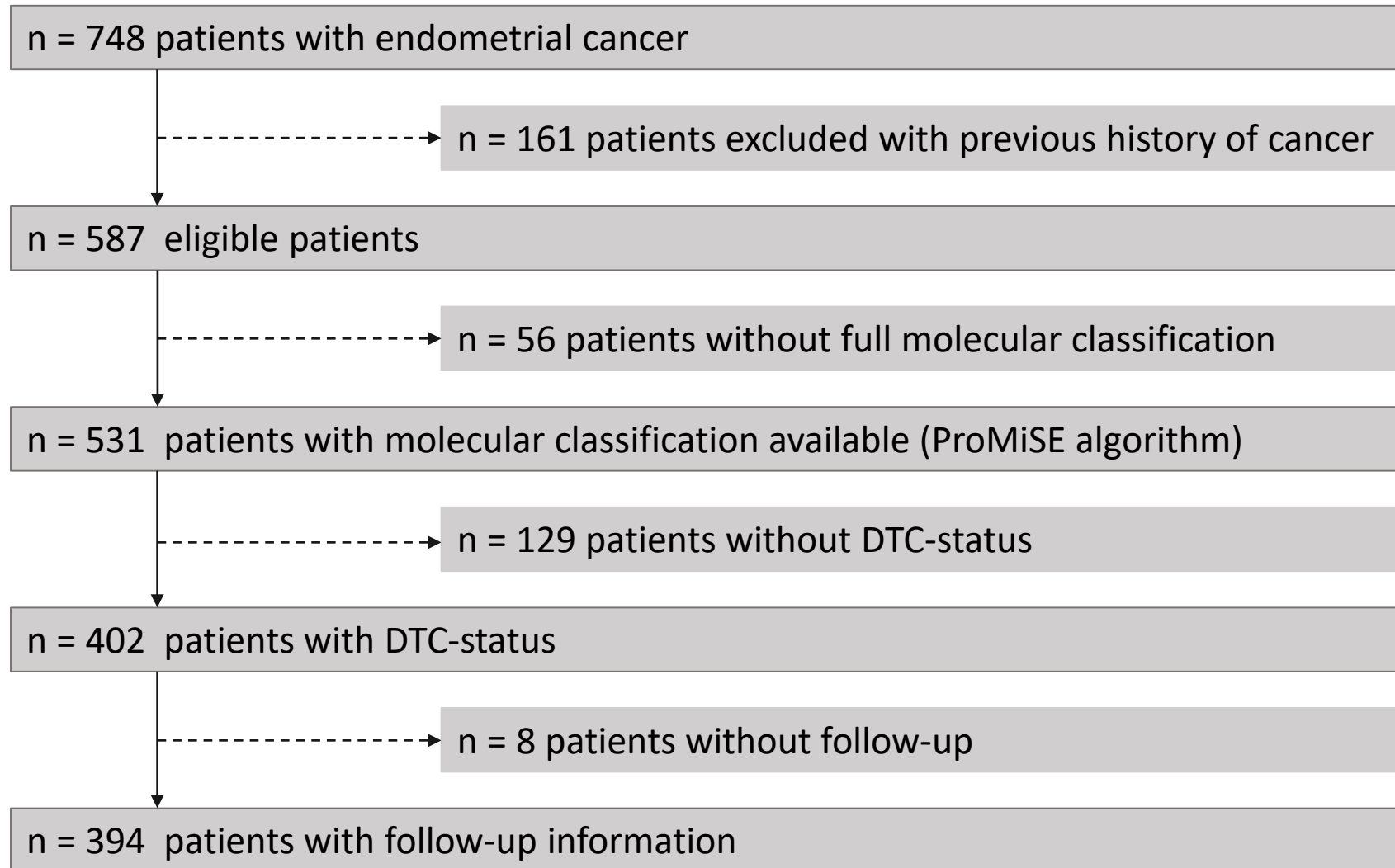

Supplement: Supplementary file 1 [file jcm-13-04489-s001.zip › Supplementary figure 1.pdf]

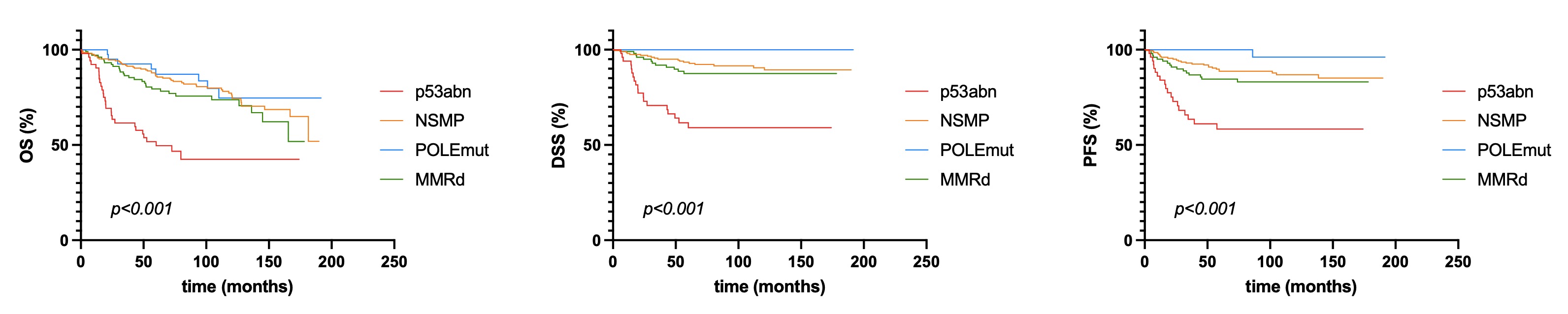

Supplement: Supplementary file 1 [file jcm-13-04489-s001.zip › supplementary figure 2.jpg]
